# Evaluation of skin phototoxicity of transdermally administered pharmaceuticals in Sprague-Dawley rats

**DOI:** 10.1186/s42826-020-00074-w

**Published:** 2020-11-19

**Authors:** Nam Hee Youn, Eun Ji Kim, Jung-Sun Yi, Joo Hwan Kim, Ye-Jin Cho, Ki Taek Nam, Ki Sook Park, Jong Kwon Lee

**Affiliations:** 1 Toxicological Evaluation and Research Department, National Institute of Food and Drug Safety Evaluation, Ministry of Food and Drug Safety, Cheongju, 28159 Republic of Korea; 2 Severance Biomedical Science Institute, Brain Korea 21 PLUS Project for Medical Science, Yonsei University College of Medicine, Seoul, 03722 Republic of Korea

**Keywords:** Phototoxicity, In vivo test, Transdermal, Skin reaction, Histopathology

## Abstract

Some drugs cause phototoxicity in humans when exposed to light, thus there is a need for an in vivo phototoxicity test to evaluate them. However, an in vivo phototoxicity test method to evaluate this has not been established. This study aimed to establish an in vivo phototoxicity test method for transdermally administered drugs. For this, we evaluated the phototoxicity using Sprague-Dawley (SD) rats for transdermal administered drugs and we studied the appropriate UVA dose using 8-methoxypsalen, which is a well-known phototoxic drug. We found that a UVA dose of 15 J/cm^2^ was dose and time dependent response compared to other UVA doses. We performed the Minimum Erythema Dose (MED) test because UVB can cause skin irritation by itself and selected 0.01 J/cm^2^ as an appropriate dose of UVB. Using the selected UVA and UVB doses, we performed a phototoxicity study of 6 pharmaceutical drugs, which included phototoxic and non-phototoxic drugs. As a result of the phototoxicity test, 100% accuracy was obtained when compared with previous studies. In addition, we performed histopathology to confirm the new findings. We found that histopathology can be used as an additional indicator of phototoxicity test for transdermally administered drugs.

## Introduction

Sunlight is comprised of visible light, infrared light, and ultraviolet light, each with a different range of wavelengths. Ultraviolet light has a shorter wavelength than visible light, has high energy, and a strong chemical action. It can cause various phototoxic reactions, including acute reactions such as erythema on the skin, as well as chronic reactions such as photoaging. Phototoxicity is mainly induced by exposure of photoreactive chemicals to ultraviolet rays [1]. These compounds are chemically activated by ultraviolet rays and bind to DNA in the cell nucleus, or act on oxygen to form oxygen radicals or singlet oxygen molecules, which affect skin cells. Skin exposed to UV rays may exhibit symptoms such as erythema, eschar formation, edema, itchiness, stinging, and in severe cases, blistering or pigmentation may occur [1].

In addition, chemicals such as psoralen, quinolone-based antibiotics, anti-inflammatory drugs, and antidepressants among pharmaceuticals are reported to induce phototoxicity. Studies such as these are important in the evaluation and regulation of phototoxicity in pharmaceuticals [2–7]. The demand for evaluation of the phototoxicity evaluation of drugs is increasing, and the International Council for Harmonisation of Technical Requirements for Pharmaceuticals for Human Use (ICH) has established the Photosafety Evaluation of Pharmaceuticals (ICH S10), which presents general considerations for internationally agreed phototoxicity evaluation in Guidance on Nonclinical Safety Studies for the Conduct of Human Clinical Trials and Marketing Authorization for Pharmaceuticals (ICH M3, R2) [8, 9]. However, ICH S10 doesn’t describe a specific method of in vivo testing for evaluation of phototoxicity, so an in vivo testing method is necessary.

Selection of appropriate UV irradiation conditions is critical for both in vitro and in vivo phototoxicity testing. UVA doses ranging from 5 to 20 J/cm^2^ are successfully used in current phototoxicity testing, according to ICH S10. UVA penetrates deeper than the epidermis and reaches the capillaries, but UVB is limited to the epidermis. Therefore, in order to testing transdermal drugs, the Minimum Erythema Dose (MED) test should be performed to select the minimum UVB dose with no erythema observed. In addition, the evaluation of in vivo phototoxicity is based on skin reaction of the skin, which is subjective and may vary depending on the tester because it relies on a gross examination method. Therefore, we suggest the selection of an appropriate dose of light for an in vivo phototoxicity test and the possibility of using histopathology as an additional indicator in evaluating phototoxicity.

## Methods/experimental

### Chemicals

The phototoxic drugs 8-Methoxy psoralen (8-MOP), Ibuprofen (IBF), Benzoyl peroxide (BPO), Ketoprofen (KPF), and Piroxicam (PXC) were used as positive substances and the non-phototoxic drug Sulisobenzone (SBZ) was used as a negative substance. A total of 6 substances were tested, and Acetone: DMSO (10:1) was used as the vehicle control (V.C). On the day of the test, test substances were prepared and the concentration of the substance was chosen as the highest concentration of soluble in vehicle control. 8-MOP, IBF, BPO, KPF, PXC, and SBZ were purchased from Sigma-Aldrich (St. Louis, MO, USA). All other reagents and materials were purchased from commercial sources.

### Animals

Six-week-old female SpragueDawley (SD) rats (135–150 g, body weight) were purchased from Koatech (Pyeongtaek, Korea) and acclimated at an animal facility in the Korea Ministry of Food and Drug Safety (Certification Number: 1501MFDS08) in accordance with the Association for Assessment and Accreditation of Laboratory Animal Care (AAALAC) International Animal Care Policies (Accredited Unit, MFDS: Unit No. 001492). The animals were acclimated for at least 5 days prior to experimentation. During the acclimation period, clinical signs were checked once every day. SD rats were housed in polycarbonate cages with free access to food and water, and maintained on a 12 h dark/light cycle in a room with controlled temperature at 22 ± 1 °C and with relative humidity of 50 ± 10%.

### Irradiation conditions

A UV irradiation device (Bio-Spectra, Vilber lourmat, Germany) equipped with a UV tube (T-40.L, Vilber lourmat, Germany) was used as a light source. The UVA lamp, emitting 320–380 nm wavelengths, was used at an intensity of 2.2 mW/cm^2^. The UVB lamp, emitting 290–320 nm wavelengths, was used at an intensity of 2.2 mW/cm^2^. In addition, the light irradiation intensity was checked using a UVP UVX radiometer (UVP, USA), UVP UVX-36 (UVP, USA), UVX-31 (UVP, USA).

### Preparation of animals

One day before the test, rats were separated into groups and shaved. Weight variation did not exceed 20% of the mean weight and the rats were randomly separated into the test groups according to body weight. Three rats per group were used, and the rat number was assigned to ‘test number-individual number’. Rats with abnormal skin lesions were excluded from the test. Rats were anesthetized intraperitoneally using sodium pentobarbital (30 mg/kg), and the hair of rats on the entire back of each rat was shaved with clippers. For convenience of observationof irradiation and phototoxic reaction, the irradiation site of the entire back of each rat was divided into 4 sites and spotted at 1.5 × 1.5 cm^2^ using a skin marker. Except for this site, the aluminum foil was used to block the light. The same procedures were performed on the non-irradiated group as were performed on the irradiated group.

### Minimum erythema dose (MED) test

The rats were irradiated with 0.01, 0.03, 0.05, 0.1, 0.15, 0.2, 0.25, 0.3, 0.35, 0.4, 0.45, 0.5 J/cm^2^ UVB. After 24, 48, and 72 h of irradiation, phototoxic skin reactions (erythema/eschar and edema) were evaluated and scored according to Draize’s method (Table 1).

**Table 1:** Evaluation of skin reactions (Draize’s Criteria)

Score for erythema/eschar formation:	
0;	No erythema
1;	Very slight erythema (barely perceptible)
2;	Well defined erythema
3;	Moderate to severe erythema
4;	Severe erythema (beet redness) to slight eschar formation (injuries in depth)
Score for edema formation:	
0;	No edema
1;	Very slight edema (barely perceptible)
2;	Slight edema (edges of area well defined by definite raising)
3;	Moderate edema (raised approximately 1 mm)
4;	Severe edema (raised more than 1 mm and extending beyond area of exposure)

### Irradiation dose selection test

This test was performed by transdermal administration of 8-MOP, which was prepared on the day of the test. We used the highest concentration of drug that was soluble in the vehicle control. The concentration of the test substance is as follows: 8-MOP 0.0001, 0.001, 0.01 w/v %. The test substances were treated 100 μl of 8-MOP solution was applied to the application site (1.5 × 1.5 cm^2^) on the back of the rat using a pipette. After 30 min of treatment with 8-MOP, UV irradiation was performed (UVA 5, 10, 15, 20 J/cm^2^; UVB 0.01 J/cm^2^). After 24, 48, 72 h of irradiation, phototoxic skin reactions (erythema/eschar and edema) were evaluated and scored according to Draize’s method (Table 1).

### Phototoxicity test of tansdermal administration by gross examination

Five positive substances (8-MOP 0.0001, 0.001, 0.01 w/v %; BPO 0.1, 1, 10 w/v %; IBF 0.1, 10, 25 w/v %; KPF 4, 40, 80 w/v %; PXC 0.1, 1, 2 w/v %) and one negative substance (SBZ 1 w/v %) were administered transdermally. The test substances were treated 100 μl of 8-MOP solution was applied to the application site (1.5 × 1.5 cm^2^) on the back of the rat using a pipette. After 30 min of substance treatment, UV irradiation was performed (UVA 15 J/cm^2^ and UVB 0.01 J/cm^2^). After 24, 48, 72 h of irradiation, phototoxic skin reactions (erythema/eschar and edema) were evaluated and scored according to Draize’s method (Table 1).

### Skin reaction evaluation

After 24, 48, 72 h of irradiation, phototoxic skin reactions (erythema/eschar and edema formation) were evaluated and scored according to Draize’s criteria (Table 1) [10]. For each test group, the skin reaction scores (erythema/ eschar and edema) of individual animals were summed for each site and the mean score was calculated according to the following equation:

Mean score = Total of erythema and edema scores/Number of animals tested [1].

A tested substance was judged to be phototoxic if the mean score of the UV-irradiated group or site was higher than that of the non-irradiated group or site at any observation period. In this study, no statistical analysis was performed.

### Histopathology of phototoxicity test

Histopathology was performed with two representative samples each of the phototoxic drug 8-MOP, BPO, and KPF tests. On the last day of the test (72 h after irradiation), the skin tissues of the rats were separated. Skin tissues were attached to thick paper in a flat orientation using a stapler, then fixed with 4% PFA, embedded in paraffin, sectioned, stained with hematoxylin and eosin (HE), and examined microscopically by a certified pathologist.

## Results

### Minimum erythema dose (MED) test

**Fig. 1:**
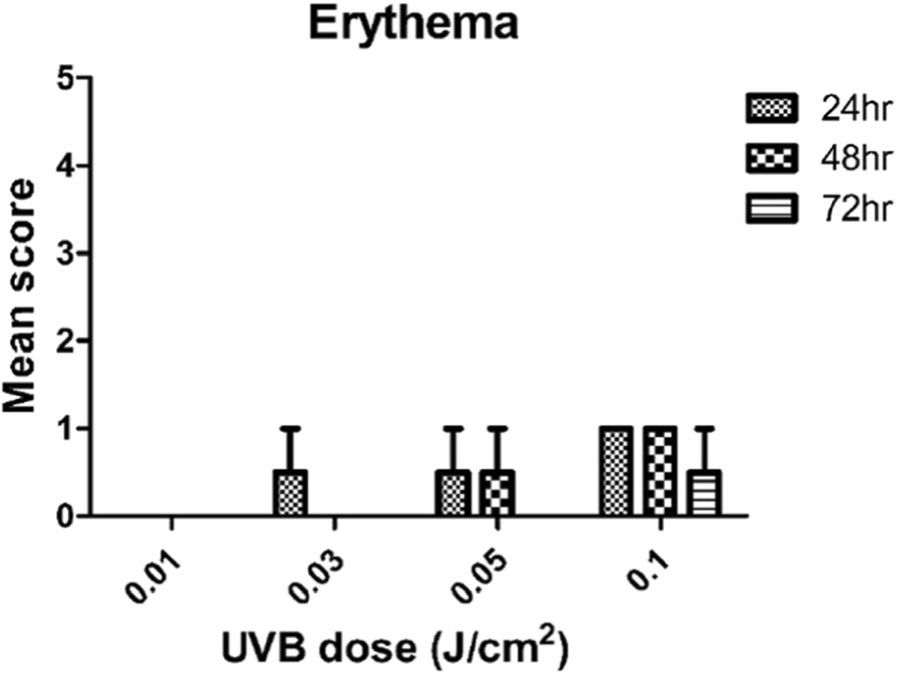
Skin score of the minimum erythema dose test after 24, 48, 72 h of UVB 0.01, 0.03, 0.05, 0.1 J/cm^2^ in SD rats. Mean score = Total of erythema and edema scores/Number of animals tested (*n* = 3)

The results of the phototoxic reaction induced by UVB in SD rats are shown in Fig. 1. Phototoxic reactions were observed at doses of 0.03, 0.05, 0.1, 0.15, 0.2, 0.25, 0.3, 0.35, 0.4, 0.45, 0.5 J/cm^2^ and no phototoxic reaction was observed at any time point at 0.01 J/cm^2^. From these results, it was confirmed that the dose of irradiation that the phototoxicity is not induced by UVB is 0.01 J/cm^2^ is the MED for UVB. Therefore, this dose was used in the irradiation dose selection test and the phototoxicity test of transdermal administration were used.

### Irradiation dose selection test

**Fig. 2:**
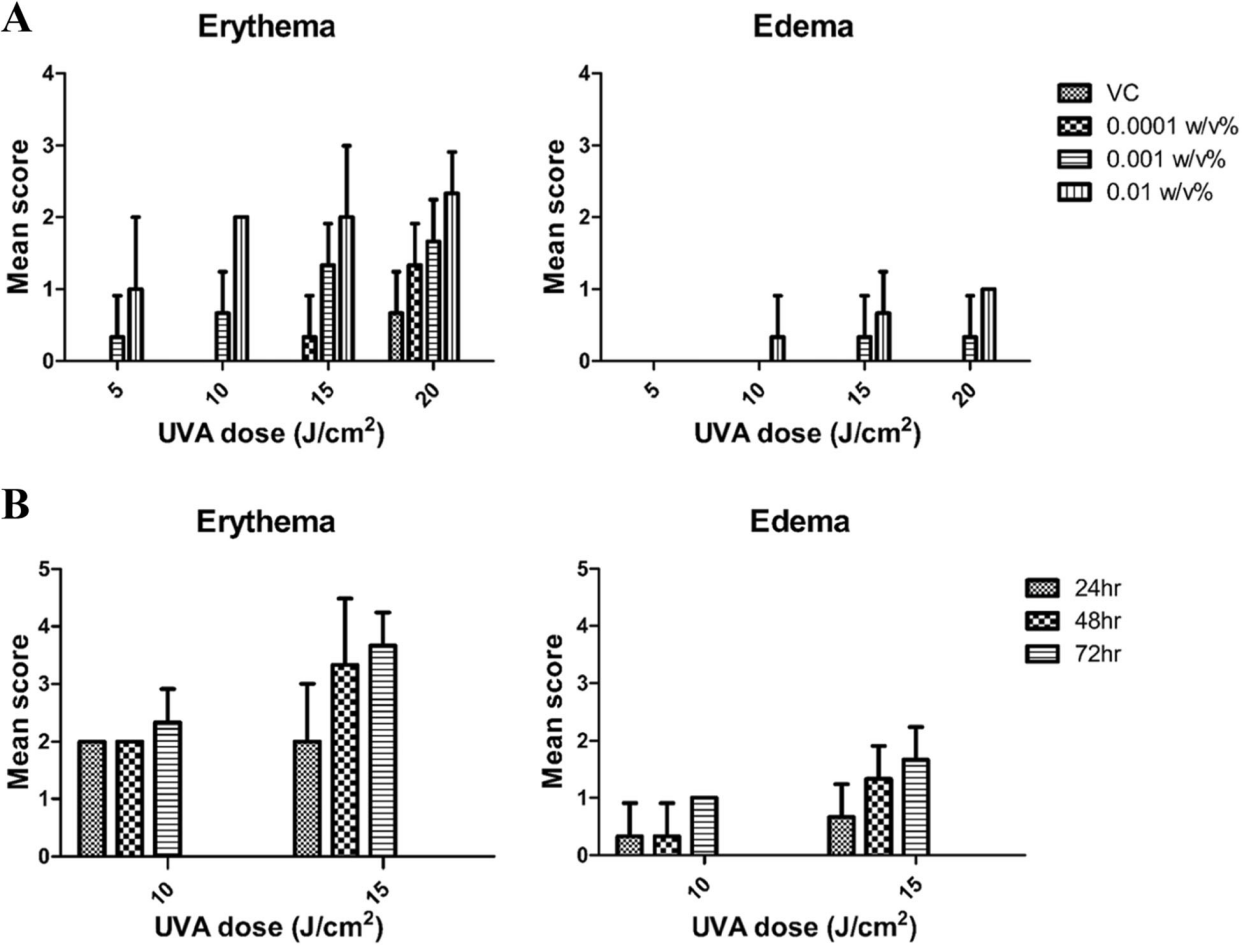
Skin score of irradiation dose selection test using 8-MOP in SD rats. **a** Dose-dependent skin reaction after 24 h of UVA 5, 10, 15, 20 J/cm^2^ administered with 8-MOP 0.01, 0.001, 0.0001 w/v %. **b** Time-dependent skin reaction after 24, 48, 72 h of UVA 10, 15 J/cm^2^ administered with 8-MOP 0.01 w/v %. Data of non-irradiated group are not shown because no have scores. Mean score = Total of erythema and edema scores/Number of animals tested (*n* = 3)

The phototoxic reactions induced by 5–20 J/cm^2^ of UVA and 0.01 J/cm^2^ of UVB in SD rats are shown in Fig. 2. As shown in Fig. 2a, erythema of the irradiated site of V. C administration was observed at 20 J/cm^2^, and edema was not observed at any concentration of 8–MOP at 5 J/cm^2^, so dose-dependent results could not be confirmed. Therefore 5 and 20 J/cm^2^ were excluded from further selection of UVA irradiation dose. As shown in Fig. 2b, the time-dependent tendency of the phototoxic reaction could not be observed at 10 J/cm^2^, and the time-dependent tendency of both erythema and edema was observed at 15 J/cm^2^. Based on these results, we selected 15 J/cm^2^ as the appropriate UVA irradiation dose to identify dose and time dependent results.

### Phototoxicity test of tansdermal administration by gross examination

**Fig. 3:**
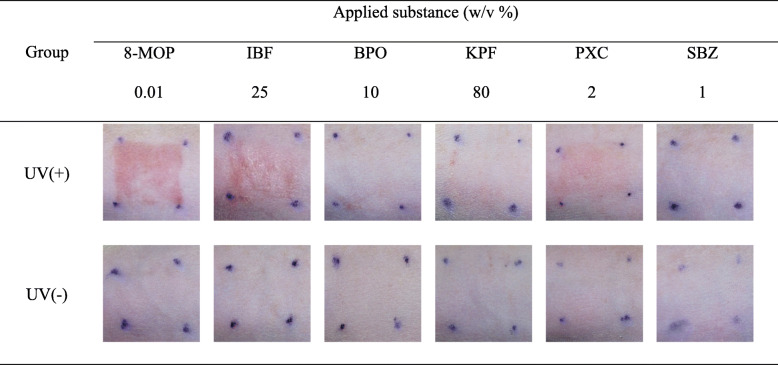
Photographs of the skin reactions after 48 h of UVA 15 J/cm^2^ and UVB 0.01 J/cm^2^ in SD rats administered with 8-MOP, IBF, BPO, KPF, PXC, SBZ. Data shows only high concentration

The phototoxic reactions induced by 8-MOP, BPO, IBF, KPF, PXC, SBZ with UVA 15 J/, UVB 0.01 J/cm^2^ irradiation in SD rats are shown in Table 2 and Fig. 3 (Data shows only high concentration). In the 8-MOP irradiation group, erythema was observed at 0.0001 and 0.001 w/v %, and erythema, eschar and edema were observed at 0.01 w/v %. In IBF (0.1, 10, 25 w/v %) and PXC (0.1, 1, 2 w/v %) irradiation group was observed erythema and edema were observed at all concentrations. In the BPO irradiation group, phototoxicity was not observed at 0.1 w/v % but erythema was observed at 1 and 10 w/v %. In the KPF irradiation group (4, 40, 80 w/v %) was observed erythema was observed at all concentrations and no phototoxic reaction was observed in the SBZ irradiation group (1 w/v %). No phototoxic reaction was observed in the non-irradiation group in all substances. These results were consistent with existing phototoxicity evaluation results, it was confirmed that it is consistent with the results of this study.

**Table 2:** Skin scores of SD rats after 48 h of UVA and UVB irradiation

Group	Test substance	Concentration (w/v %)	Mean score	
			erythema	edema
UV(+)	8-MOP	0.01	2.3	1.3
	IBF	25	1.6	0.3
	BPO	10	0.6	0
	KPF	80	1.3	0
	PXC	2	1.6	0
	SBZ	1	0	0
UV(−)	8-MOP	0.01	0	0
	IBF	25	0	0
	BPO	10	0	0
	KPF	80	0	0
	PXC	2	0	0
	SBZ	1	0	0

There were three SD rats per group. No phototoxic response (erythema, edema) was observed in the non-irradiation site at all time

### Histopathology of phototoxicity test

**Fig. 4:**
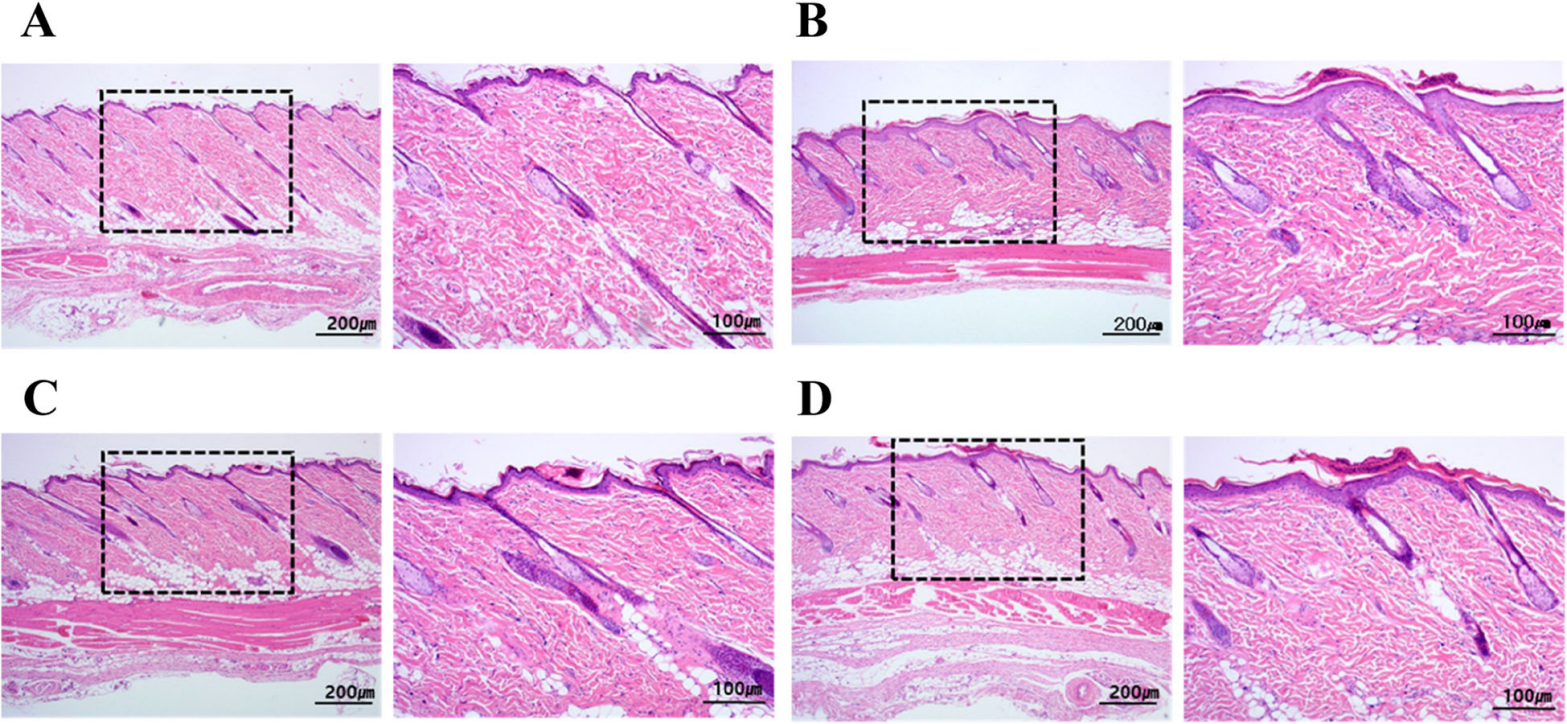
Representative histopathological photographs of SD rat skin reactions after 72 h of UVA 15 J/cm^2^ and UVB 0.01 J/cm^2^. **a** V. C irradiation site. **b** 8-MOP 0.01 w/v % irradiation site. **c** BPO 10 w/v % irradiation site. **d** KPF 80 w/v % irradiation site

Representative histopathological photographs of two phototoxic drugs (BPO and KPF) are shown in Fig. 4. Table 3 (Data shows only high concentration) summarizes the findings. In the case of BPO and KPF, which caused reactions that were ambiguous by gross examination, histopathology was performed to obtain accurate results, and representative phototoxic substance 8-MOP and V. C were also performed. In the V. C irradiation group, histopathology showed no phototoxic reaction so we observed unusual histopathological lesions. In the 8-MOP irradiation group, the phototoxic reaction was observed in the irradiated rats that received low and medium concentrations. However, at high concentrations of 8-MOP, the epidermis was thickened due to the proliferation of the epidermal layer, and showed necrosis of the stratum corneum. In the BPO irradiation group, thickening of the epidermis was observed at low concentrations, and necrosis of the stratum corneum was observed at high concentrations. In the KPF irradiation group, necrosis of the stratum corneum was observed at low, medium, and high concentrations. In the 8-MOP, BPO, and KPF treated irradiated groups showed normal histopathological lesions at all concentrations. Therefore 8-MOP, BPO, and KPF were judged to be phototoxic drugs.

**Table 3:** Summary of the phototoxicity test

Test substance	CAS.NO	Clinical^a^	Judgement	
			gross examination	Histopathology
8-MOP	298–81-7	Phototoxic	Phototoxic	Phototoxic
IBF	15,687–27-1	Phototoxic	Phototoxic	Phototoxic
BPO	94–36-0	Phototoxic	Weak-phototoxic	Phototoxic
KPF	22,071–15-4	Phototoxic	Weak-phototoxic	Phototoxic
PXC	36,322–90-4	Phototoxic	Phototoxic	Phototoxic
SBZ	4056-45-6	Non-phototoxic	Non-phototoxic	Non-phototoxic

^a^Data from Yonezawa et al. 2015 [1]

## Discussion

In this study, in order to selected the appropriate UV irradiation dose with an in vivo phototoxicity test and confirmed the phototoxicity of various drugs by two skin reaction evaluation methods. The UV irradiation dose suggested by ICH S10 is 5–20 J/cm^2^, and the irradiation dose used by other research groups varies. Some UVA values used in other studies are 10.2 J/cm^2^ for guinea pigs [11], 10 J/cm^2^ for guinea pigs [12], 30 J/cm^2^ for SD rats [13–15], and 10 J/cm^2^ for SD rats [1]. Some UVB values used in other studies are 0.3 J/cm^2^ for guinea pigs [12], 0.18 J/cm^2^ for guinea pigs [16], 0.25 J/cm^2^ for guinea pigs and 0.031 J/cm^2^ for SD rats [1].

In order to select appropriate UVA and UVB doses to use in our phototoxicity test, an irradiation dose selection test (for UVA) and a MED test (for UVB) were performed. We found that the appropriate irradiation dose of UVA, dose and time-dependent response was 15 J/cm^2^. As a result of the MED test, we found that the appropriate irradiation dose of UVB dose not causing phototoxic reaction was 0.01 J/cm^2^.

An in vivo transdermal phototoxicity test was performed using six test substances at the selected doses of irradiation. Phototoxic reactions were observed with the five positive drugs (8-MOP, BPO, IBF, KPF, PXC) and judged as phototoxic drugs. No phototoxic reactions were observed with the negative control drug (SBZ) and judged as non-phototoxic drug. Phototoxic reactions (erythema, eschar, edema) was observed and scored according to Draize’s criteria (Table 1) [1, 16, 17].

In addition, we performed histopathology as an additional indicator of the phototoxicity test. We found histopathological lesions, caused by drugs, in the skin of rats treated with 8-MOP, BPO, and KPF. Thus, we confirmed the possibility of using histopathology as an additional indicator to supplement gross examination, which is the subjective reading of the phototoxicity test. From this results, the proposed photosafety evaluation on the basis of the in vivo phototoxicity for transdermal drugs.

Recently, interest in the photosafety of drugs has increased in both regulatory agencies and industry, and regulatory agencies have recommended the implementation of the 3Rs principle (refinement, reduction, and replacement). Considering these trends, the proposed test method would be useful for evaluating the in vivo phototoxicity of drugs.

## Conclusions

Through this study, we found that suitable the irradiation doses in SD rats for testing the phototoxicity of drugs were UVA 15 J/cm^2^ and UVB 0.01 J/cm^2^. In addition, we showed that the use of histopathology as an additional indicator in the evaluation of phototoxicity allows more accurate and objective judgment. This confirmed the availability of the transdermal drug in vivo photosafety test method.

## Data Availability

Available.

## Abbreviations

8-MOP: 8-Methoxypsoralen
IBF: Ibuprofen
BPO: Benzoyl peroxide
KPF: Ketoprofen
PXC: Piroxicam
V.C: vehicle control
SD: Sprague-Dawley
UV: Ultraviolet
MED: Minimum Erythema Dose
